# Dry Etching Performance and Gas-Phase Parameters of C_6_F_12_O + Ar Plasma in Comparison with CF_4_ + Ar

**DOI:** 10.3390/ma14071595

**Published:** 2021-03-24

**Authors:** Nomin Lim, Yeon Sik Choi, Alexander Efremov, Kwang-Ho Kwon

**Affiliations:** 1 Department of Control and Instrumentation Engineering, Korea University, Sejong 30019, Korea; nomin_lim@korea.ac.kr (N.L.); choiyeonsik94@korea.ac.kr (Y.S.C.); 2 Department of Electronic Devices & Materials Technology, State University of Chemistry & Technology, 7 Sheremetevsky av., 153000 Ivanovo, Russia; amefremov@yandex.ru

**Keywords:** global warming potential, C_6_F_12_O-containing plasma, Si and SiO_2_ etching rates, plasma diagnostics, reaction kinetics, polymerization

## Abstract

This research work deals with the comparative study of C_6_F_12_O + Ar and CF_4_ + Ar gas chemistries in respect to Si and SiO_2_ reactive-ion etching processes in a low power regime. Despite uncertain applicability of C_6_F_12_O as the fluorine-containing etchant gas, it is interesting because of the liquid (at room temperature) nature and weaker environmental impact (lower global warming potential). The combination of several experimental techniques (double Langmuir probe, optical emission spectroscopy, X-ray photoelectron spectroscopy) allowed one (a) to compare performances of given gas systems in respect to the reactive-ion etching of Si and SiO_2_; and (b) to associate the features of corresponding etching kinetics with those for gas-phase plasma parameters. It was found that both gas systems exhibit (a) similar changes in ion energy flux and F atom flux with variations on input RF power and gas pressure; (b) quite close polymerization abilities; and (c) identical behaviors of Si and SiO_2_ etching rates, as determined by the neutral-flux-limited regime of ion-assisted chemical reaction. Principal features of C_6_F_12_O + Ar plasma are only lower absolute etching rates (mainly due to the lower density and flux of F atoms) as well as some limitations in SiO_2_/Si etching selectivity.

## 1. Introduction

Recently, silicon-based electronics still play the leading role in the worldwide production of integrated electronic circuits. The main materials composing such devices are the silicon itself as well as the silicon dioxide that mostly appears as a rather thin functional layer on the Si substrate [1,2,3]. The latter found numerous applications as gate insulator in various field-effect devices, spacer dielectric, passivating coating, and hard masks featured by the high stability in respect to aggressive etchant environments [1,3,4,5]. Obviously, as most of the devices have complicated multi-layer structures, the corresponding fabrication process needs the precision patterning of both Si and SiO_2_ materials. Recently, strong requirements to devise both dimension and performance are satisfied by the “dry” etching techniques, and namely by the reactive-ion etching (RIE) method [4,5]. The main feature of RIE is the simultaneous action of two parallel etching mechanisms, such as physical sputtering and ion-assisted chemical reaction [5,6]. This provides the effective adjustment of output process characteristics (etching rate, etching profile, selectivity in respect to mask and/or under-layer material, etching residues, surface roughness, etc.) by an appropriate choice of working gas and processing conditions [2,3,4,5].

The widely-used gases for reactive-ion etching of all silicon-based materials are fluorocarbon compounds with a general formula of C_x_H_y_F_z_ mixed with Ar and/or O_2_ [4,5,6]. Accordingly, there were many experimental studies (for example, Refs. [7,8,9,10,11,12,13,14,15,16,17] and earlier ones included in monographs [1,2,3,4]) reported on RIE kinetics and mechanisms for Si and SiO_2_ in various fluorocarbon gas plasmas, including CF_4_-based gas mixtures. The most important findings may be summarized as follows:

(1) The dominant role in the chemical etching pathway for Si and SiO_2_ under typical RIE conditions (p < 50 mTorr, ion bombardment energy ~200–400 eV) belongs to F atoms [5]. Fluorocarbon gases with z/x < 3 (where z and x are coefficients in the C_x_H_y_F_z_ formula) exhibit high polymerization ability that results in the deposition of fluorocarbon polymer film on the treated surface. This lowers absolute etching rates but results in the highly-anisotropic etching of silicon (due to the passivation of side walls by the fluorocarbon polymer layer) as well as in advanced SiO_2_/Si etching selectivity (due to different thicknesses of polymer films on oxygen-free and oxygen-containing surfaces) [8,9,11].
(2) Both etching and polymerization kinetics may be effectively adjusted by mixing of fluorocarbon gas with Ar and/or O_2_ [10,13,14,15,16]. Corresponding mechanisms do work through changes in both gas-phase chemistry (formation/decay balance for F atoms and polymerizing radicals) [5,17] and heterogeneous processes kinetics (physical and chemical decomposition of the fluorocarbon polymer film) [14,15,16,17].
(3) The chemical interaction of F atoms with Si and SiO_2_ exhibits different mechanisms and thus, may be controlled by different limiting stages. In the case of Si, spontaneous chemical reaction mostly produces the high volatile SiF_4_ [4,5]. That is why the Si + F reaction rate in non- or low-polymerizing plasmas is characterized by low sensitivity to the intensity of ion bombardment as well as exhibits the nearly exponential dependence on surface temperature [2,3]. Oppositely, the SiO_2_ + F reaction has the sufficient threshold energy (as the Si-O bond of ~799 kJ/mol is stronger than the Si-F of ~552 kJ/mol [18]) and occurs only as the ion-assisted process. The role of ion bombardment includes the production of adsorption sites for F atoms and the sputtering of low volatile non-saturated SiF_x_ compounds [5,7]. At the same time, ion energies above ~200 eV are generally enough to provide the reaction-rate-limited etching regime controlled by the F atom flux [7,19].

The serious problem of all fluorocarbon gases used for Si and SiO_2_ RIE processes is their destructive effect on the ozone layer and thus, high global warming potentials (GWP). For example, the GWP index for CF_4_ is over 5000 [20,21] which means its global warming impact is much higher compared with CO_2_. The increasing attention to the environmental pollution issue has motivated intensive studies of eco-friendly dry etching processes [22,23]. Particularly, it was suggested to substitute conventional process chemistries based on high-GWP fluorocarbon gases for alternate ones with lower environmental impacts [24,25]. One of the possible candidates here is the dodecafluorooxepane (C_6_F_12_O) which exhibits low GWP index of 1, has the extremely low toxicity, as well as is featured by the boiling point above the room temperature [26]. Obviously, the last property provides the much easier trapping of remaining gas from the output gas flow compared with conventional gaseous fluorocarbons as well as promotes its recovery procedure. In our previous work [27], we investigated C_6_F_12_O and CHF_3_ as additive components in the CF_4_ + O_2_ gas mixture in respect to the SiON etching process. In these experiments, the CF_4_ was subsequently substituted for one of the above gases, and the SiON etching characteristics were compared. According to this work, most important differences between CHF_3_ and C_6_F_12_O gases are that the latter (a) provides lower absolute etching rate together with higher etching selectivity over Si; (b) exhibits a bit lower increase in the polymerizing ability; and (c) produces more vertical sidewalls. At the same time, it is clear that the above study has some limitations in respect to both completeness and usability of corresponding data. First, the main focus of Ref. [27] was to compare C_6_F_12_O with CHF_3_, but not with CF_4_. At the same time, the latter is also the widely-used high-GWP gas which is waiting for an adequate low-GWP replacement. Even if this work illustrates some correlations between etching performances of C_6_F_12_O and CF_4_, these always correspond to O_2_-containing gas mixtures and cover only one combination of gas pressure and input power. Since the presence of oxygen sufficiently influences plasma parameters and densities of active species in fluorocarbon gas plasmas [5,15,17], these data say nothing about key properties of pure C_6_F_12_O and CF_4_ gases. Obviously, such situation does not help to understand features of corresponding etching processes in oxygen-free gas systems. Second, Ref. [27] mainly discussed the etching characteristics for SiON, but not for the widely used Si and SiO_2_. At the same time, the SiO_2_/Si etching selectivity is the quite important parameter for many etching processes [1,2,3]. Thirdly, Ref. [27] has a rather phenomenological nature and thus, did not discuss differences between two gas systems in the light of gas-phase plasma parameters and plasma chemistry. Such a situation in some extent lowers the significance of corresponding results, since those seem to be valid only for a given combination of processing gases and treated materials. Therefore, unknown relationships between processing conditions, gas-phase plasma parameters, and RIE kinetics do not allow evaluating real perspectives of C_6_F_12_O as an etchant gas for silicon-based materials. Obviously, this retards the development of environmentally-friendly dry etching technologies.

The main idea of this work was to compare reactive-ion etching performances for low-GWP C_6_F_12_O + Ar and high-GWP CF_4_ + Ar plasmas in respect to Si and SiO_2_ with a focus on effects of gas pressure and input power. When making a decision on gas mixtures, we wanted to test the C_6_F_12_O as the individual chemical etchant as well as to perform a comparison with the quite simple and the well-studied gas system, such as CF_4_. The absence of O_2_ in both feed gases provides a chance to attribute some specific etching effects (if those do exist) to the presence of oxygen in the C_6_F_12_O molecule. The choice of Ar as an additive component was because of its wide use in real etching processes in a combination with fluorocarbon gases [1,2]. The general aims are to stabilize plasma at low pressures, to minimize polymerization-related effects, as well as to reduce the amount of fluorocarbon compounds in the output gas flow [3,4,5]. Accordingly, the main goals were: (1) to analyze features of corresponding etching processes in terms of etching rate, etching selectivity, and residues; (2) to study interconnections between processing parameters and gas-phase plasma characteristics (electron temperature, energy of ion bombardment, densities and fluxes of plasma active species); and (3) to formulate conclusions on differences and/or similarities of etching mechanisms for Si and SiO_2_ in given gas systems. Another important issue is that we studied the low input power etching regime with ~10 times lower plasma density compared with the conventional RIE process. As was shown in Refs. [28,29], the latter provides weaker surface damage due to lower ion flux as well as exhibits more anisotropic etching because of reduced neutral/charged ratio.

## 2. Materials and Methods

### 2.1. Experimental Setup and Procedures

**Figure 1:**
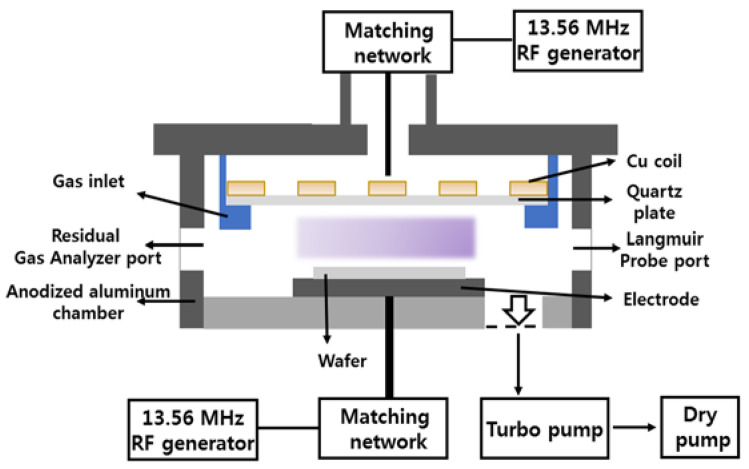
Schematic diagram of the reactor chamber with surroundings.

Both etching and plasma diagnostics experiments were performed in the planar inductively coupled plasma (ICP) reactor known from our previous studies [17,30]. Schematic diagram of reactor chamber with arrangements is shown in Figure 1. Plasma was excited using the 13.56 MHz power supply while another 13.56 MHz rf generator powered the bottom electrode in order to control the ion bombardment energy through the bias power ($W_{dc}$). Initial compositions of CF_4_ + Ar and C_6_F_12_O + Ar gas mixtures were set by equal partial flow rates of component gases within the total flow rate of 40 sccm. As such, each gas mixture was always composed by 50% of argon and 50% of one of fluorocarbon components. Variable processing parameters were the input RF power (W = 200–600 W that corresponded to the input power density of about 0.02–0.06 W/cm^3^) and the gas pressure (p = 4–12 mTorr). In addition, the constant bias power $W_{dc}$ = 200 W produced the variable negative dc bias voltage ($-U_{dc}$), according to the change in positive ion flux. The parameter $-U_{dc}$ was measured using the high-voltage probe (AMN-CTR, Youngsin-RF Co. Ltd, Seoul, Korea). 

Plasma diagnostics by the double Langmuir probe (DLP2000, Plasmart Inc., Daejeon, Korea) provided the data on electron temperature ($T_{e}$) and ion current density ($J_{+}$). The treatment of measured I–V curves accounted for well-known concepts of Langmuir probe theory for low pressure plasmas [31]. In order to minimize experimental errors due to the deposition of fluorocarbon polymer on probe tips, the latter were cleaned in 50% Ar + 50% O_2_ plasma before and after each measurements. Our previous works have demonstrated the efficiency of such procedure to obtain adequate diagnostics results in polymerizing fluorocarbon-based plasmas [7,30,32,33,34].

Plasma diagnostics by optical emission spectroscopy (AvaSpec-3648, JinYoung Tech, Seoul, Korea) was applied to compare F atom densities in C_6_F_12_O + Ar and CF_4_ + Ar plasmas as well as to trace behaviors of both $n_{F}$ and $n_{O}$ with variations in processing conditions. For this purpose, we monitored emission intensities (I) for three atomic lines, such as Ar 750.4 nm, F 703.8 nm, and O 777.0 nm. These are widely used actinometrical lines which are characterized by (a) direct electron impact excitation mechanism; (b) low lifetimes of corresponding excited states that allows one to neglect their non-radiative relaxation; and (c) known actinometrical coefficients for couples of F 703.8 nm/Ar 750.4 nm ($C_{act}^{F}$) and O 777.0 nm/Ar 750.4 nm $C_{act}^{O}$ [35]. Accordingly, one can write $n_{X}/n_{Ar}=\left(I_{X}/I_{Ar}\right)C_{act}^{X}$ (where *X* = F or O) and then, use the $n_{X}/n_{Ar}$ ratio to find the fraction of target particle $y_{X}$ by assuming $y_{Ar}$ = 0.5. The estimation of absolute densities was impossible due to unknown gas temperature.

Etching kinetics for Si and SiO_2_ was studied using fragments of Si (111) wafers without or with oxide layer. Both samples were simultaneously placed in the middle part of the bottom electrode. The latter had a built-in water-flow cooling system that allowed one to maintain its temperature at the nearly constant value of ~17 °C within the processing times *τ* ~5 min. The sample size of ~2 × 2 cm allowed one to neglect the loading effect as well as to provide the etching regime controlled by heterogeneous process kinetics. Preliminary experiments indicated no principal (i.e., exceeding the standard experimental error) differences in I–V curves measured with and without sample loading. Therefore, one can neglect the sensitivity of gas-phase plasma parameters to etching products as well as consider the gas phase to be the permanent source of active species. In order to determine etching rates, we developed a partial surface masking by the photoresist AZ1512 with a thickness of ~1.5 µm as well as measured etched depths Δh for the processing time τ = 1 min using the surface profiler Alpha-Step 500 (Tencor, Milpitas, CA, USA). The quasi-linear shape of Δh=f(τ) curves in both gas mixtures surely suggests the steady-state etching regime characterized by the time-independent etching rate R. As such, the latter may be simply calculated as R=Δh/τ.

The chemical compositions of plasma-treated Si and SiO_2_ surfaces were examined using X-ray photoelectron spectroscopy (K-Alpha, Thermo VG, UK) with a monochromatic Al K_α_ source (1486.6 eV). The size of the X-ray beam was 200 µm, and the electron emission angle was 45 degrees. The acceleration voltage and emission current in the X-ray source were 12 kV and 3 mA, respectively. The base pressure in the XPS chamber was 2.9 × 10^−9^ mbar, and the operating pressure was maintained at 4.9 × 10^−9^ mbar. A Flood gun was used for charge compensation. In order to keep the real etched surface condition, we did not perform the Ar^+^ sputtering procedure before the analysis.

### 2.2. Approaches for the Analysis of Etching Kinetics

For the phenomenological analysis of Si and SiO_2_ etching kinetics, one can account for known features of the reactive-ion etching process in fluorocarbon-based plasmas [5,9,11,13,17,30,32,33,34,36,37]. These are as follows:

(1) Under typical reactive-ion etching conditions (p < 50 mTorr and $-U_{dc}$ > 200 V that provide an excess of ion bombardment energy over sputtering thresholds [3,4,5,6] for target materials), the experimentally obtained etching rate R is the superposition of two parallel etching pathways, such as physical sputtering and ion-assisted chemical reactions. Accordingly, one can simply suggest $R=R_{phys}+R_{chem}$ [5,36,37].
(2) The rate of physical sputtering, $R_{phys}$, may be found as $Y_{S}\Gamma_{+}$ [5,36], where $Y_{S}$~$\sqrt{\varepsilon_{i}}$ [30,32,33,34] is the sputtering yield, $\varepsilon_{i}=\left|-U_{f}-U_{dc}\right|$ is the ion bombardment energy, $-U_{f}\approx 0.5T_{e}\ln\left(m_{i}/2\pi m_{e}\right)$ is the floating potential, and $\Gamma_{+}\approx J_{+}/e$ is the flux of positive ions. As such, the relative change in $R_{phys}$ with variations of processing conditions may be traced by the parameter $\sqrt{\varepsilon_{i}}\Gamma_{+}$ characterizing the ion momentum flux [32,33,34].
(3) The rate of ion-assisted chemical reaction, $R_{chem}$, is represented by the multiplication of $\gamma_{R}\Gamma_{F}$ [17,30,34], where $\gamma_{R}=s_{0}\left(1-\theta \right)$ [17] is the effective reaction probability, $s_{0}$ is the sticking probability for etchant species on the free adsorption site, and θ is the fraction of adsorption sites occupied by reaction products, and $\Gamma_{F}$ is the thermal flux of F atoms with the gas-phase density of $n_{F}$. In general case, the situation θ → 0 corresponds to the reaction-rate-limited process regime where $\gamma_{R}\approx s_{0}$ is only the exponential function of surface temperature. Oppositely, the condition θ → 1 points out on the ion-flux-limited process regime. Here, even if the nearly constant surface temperature provides $s_{0}$ ≈ const, the trend of $R_{chem}$ is controlled by the change in $\gamma_{R}$ through the fraction of free adsorption sites for F atoms (1−θ). In polymerizing plasmas, $\gamma_{R}$ may also be sensitive to fluorocarbon polymer thickness if the latter provides $\Gamma_{F}{}^{\prime }/\Gamma_{F}$ << 1, where $\Gamma_{F}{}^{\prime }$ is the flux of F atoms on the polymer film/etched surface interface [5,9,13].

## 3. Results and Discussion

**Figure 2:**
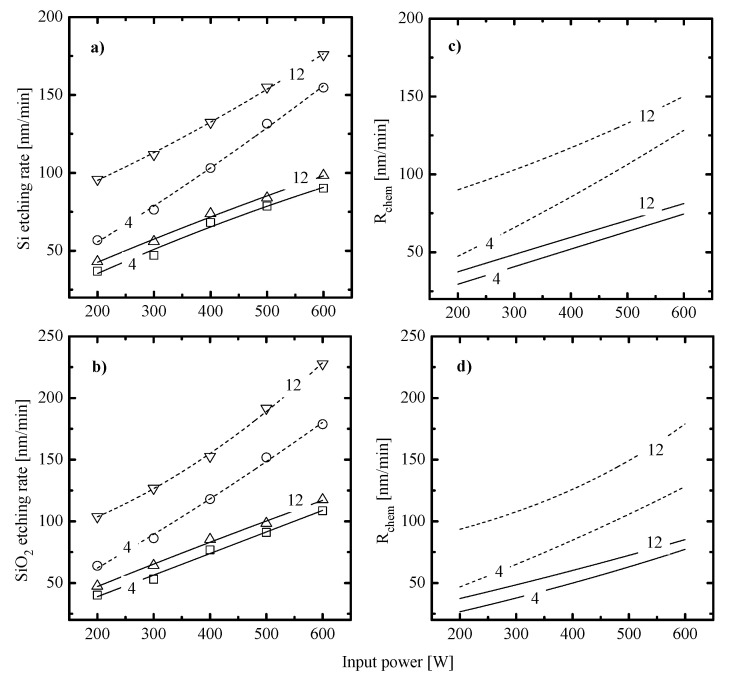
Measured etching rates (**a**,**b**) and calculated rates of ion-assisted chemical reaction (**c**,**d**) for Si and SiO_2_ in 50% C_6_F_12_O + 50% Ar (solid lines) and 50% CF_4_ + 50% Ar (dashed lines) plasmas. Numerical labels on curves mean the gas pressure in mTorr.

**Figure 3:**
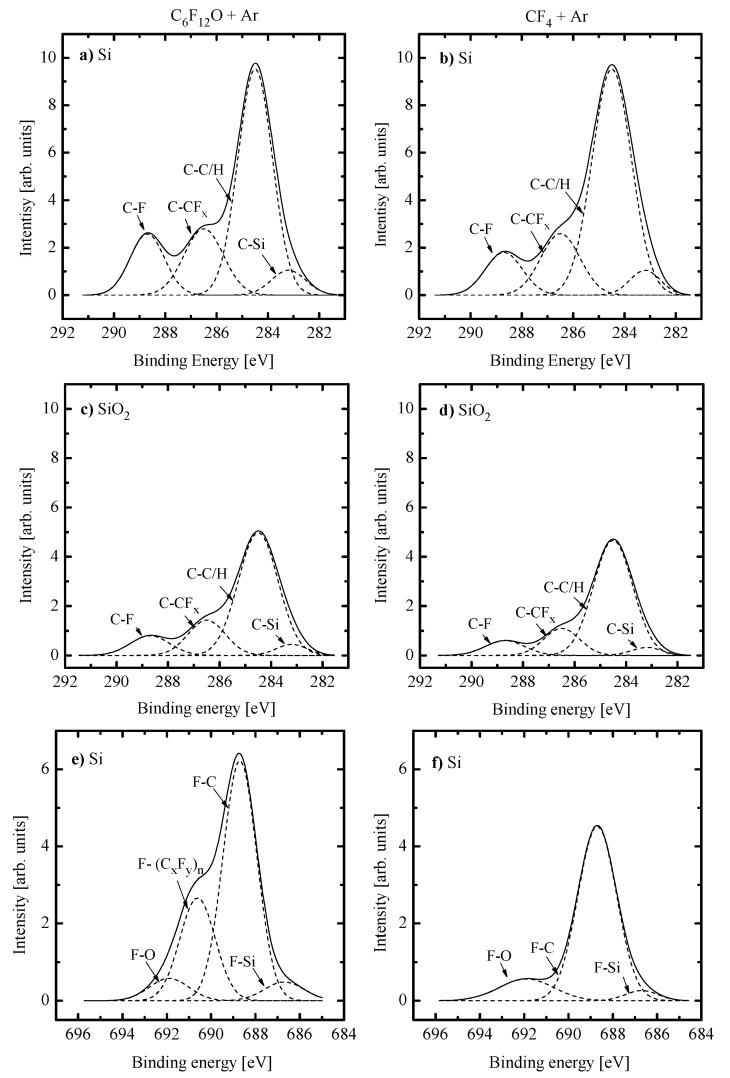
C(1s) (**a**–**d**) and F(1s) (**e**,**f**) XPS spectra for Si (**a**,**b**,**e**,**f**) and SiO_2_ (**c**,**d**) surfaces etched in 50% C_6_F_12_O + 50% Ar (**a**,**c**,**e**) and 50% CF_4_ + 50% Ar (**b**,**d**,**f**) plasmas. Processing condition are W = 400 W, p = 8 mTorr.

Figure 2a,b illustrate effects of input power and gas pressure on Si and SiO_2_ etching rates in 50% C_6_F_12_O + 50% Ar and 50% CF_4_ + 50% Ar plasmas. It can be seen that both Si and SiO_2_ are characterized by similar monotonic R=f(W) at p = const curves as well as exhibit higher etching rates at higher pressures. The main peculiarity here is only that the C_6_F_12_O + Ar gas system provides the systematically lower absolute etching rates (by ~1.5–1.6 times for p = 4 mTorr and ~1.8–2.0 times for p = 12 mTorr at 200–600 W). The similar difference was obtained in Ref. [27] for C_6_F_12_O + O_2_ and CF_4_ + O_2_ plasmas. The analysis of these data with accounting for known features of Si and SiO_2_ etching mechanisms in fluorocarbon gas plasmas ([5,7,8,9,10,11,12,13,14,15,16,17], see Section 1) allows one to conclude that:

(1) Similar changes of etching rate for each material in C_6_F_12_O- and CF_4_-based plasmas vs. input power and gas pressure may be attributed to similar etching regimes. In general, this may be either the polymer-thickness-controlled etching process (through the transport of etchant species to the film/etched surface interface) or the chemical reaction itself under the condition of thin or even non-continuous polymer film. In our case, the second variant looks more favorable because of the low polymerizing ability of CF_4_ plasma [3,4,5,7,8,17] as well as the similar feature of the C_6_F_12_O + Ar gas system. As follows from Figure 3a–d, the latter exhibits the only small increase in the amount of carbon-containing compounds on both Si and SiO_2_ surfaces. In addition, one can see that both gas systems provide the lower amount of fluorocarbon polymer on SiO_2_ compared with that on Si. This well-known effect is due to the etching of polymer by O atoms on the film/SiO_2_ interface [5,6,7,8].
(2) Similar changes of Si and SiO_2_ etching rates in each gas system indicate that corresponding chemical etching pathways are driven by identical active species and have one and the same limiting stage. In particular, an increase in both Si and SiO_2_ etching rates vs. gas pressure surely points out on the absence of ions-driven limiting stages in corresponding heterogeneous reaction schemes. In fact, this means that the SiO_2_ + F reaction kinetics is not limited by the ion-assisted destruction of oxide bonds SiO_x_(s.) → Si(s.) + xO to produce adsorption sites for F atoms. Probably, such situation is due to the quite high ion bombardment energy used in this study. As such, all experimental curves in Figure 2a,b reflect changes in the Si(s.) + xF → SiF_x_ reaction kinetics while systematically lower etching rates in C_6_F_12_O + Ar plasma may preliminary be attributed to corresponding differences in gas-phase densities of F atoms.

**Figure 4:**
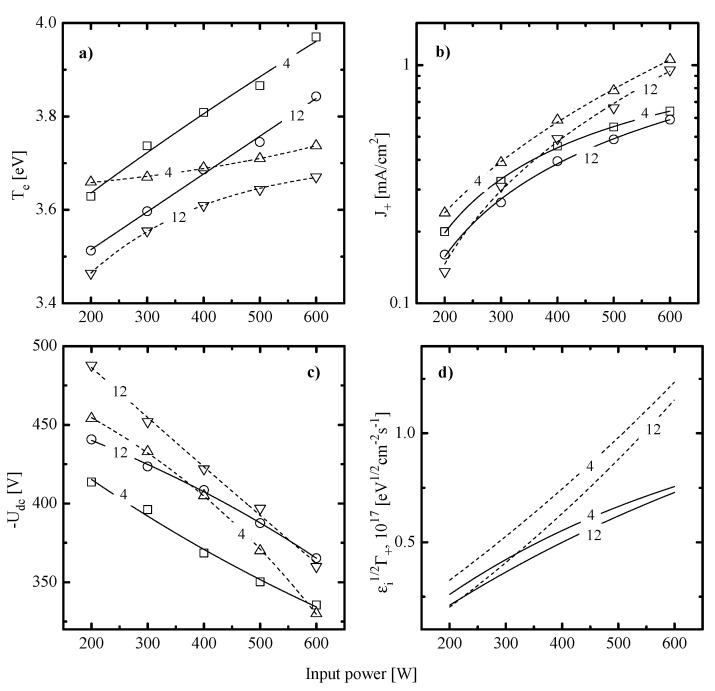
Electron- and ion-related plasma parameters in 50% C_6_F_12_O + 50% Ar (solid lines) and 50% CF_4_ + 50% Ar (dashed lines) plasmas: (**a**) electron temperature; (**b**) ion current density; (**c**) negative dc bias at constant $W_{dc}$ = 200 W; and (**d**) parameter $\sqrt{\varepsilon_{i}}\Gamma_{+}$ characterizing the ion momentum flux. Numerical labels on curves mean the gas pressure in mTorr.

In order to check the above suggestions on etching mechanisms as well as to analyze differences between two gas systems, the data on plasma parameters and densities of active species are needed. From Figure 4, it can be seen that C_6_F_12_O + Ar and CF_4_ + Ar plasmas exhibit similar behaviors for electrons- (electron temperature, electron density) and ions- (ion flux, ion bombardment energy) related gas-phase parameters. Most of the obtained effects are in agreement with those for many other fluorocarbon gas plasmas [17,30,32,33,34] and may briefly be commented as follows:

- The electron temperature (Figure 4a) increases toward higher input powers at p = const and decreases toward higher pressures at W = const. The first phenomenon is probably due to a decrease in electron energy losses for vibrational and electronic excitations of dominant neutral particles. The evident reason is an increase in densities of less saturated radicals and atomic species due to the acceleration of electron-impact dissociation for multi-atomic components. The faster growth of $T_{e}$ in the C_6_F_12_O + Ar plasma as well as higher electron temperatures at W > 400 W may result from the multichannel fragmentation mechanism with including C_x_F_y_ + O/O(^1^D) → C_x-n_F_y-m_O + C_n_F_m_ and CF_x_ + O/O(^1^D) → CF_x-1_O + F reactions [32,33]. As such, one can easily imagine the situation when the given gas system provides higher densities of less saturated species compared with CF_4_ + Ar under identical processing conditions. The decreasing tendency for $T_{e}=f\left(p\right)$ is surely connected with an increase in the overall electron energy loss due to increasing electron-neutral collision frequency [5].
- The ion current density (Figure 4b) in both gas systems mainly reflects the change of $n_{+}$ and thus, depends on the positive ion formation/decay balance. Particularly, an increase in W at p = const surely results in increasing total ionization rates and thus, causes the same response from the side of $n_{+}$. It should be noted that a bit lower $J_{+}$ values in the C_6_F_12_O + Ar plasma at W > 400 W are in formal agreement with above suggestion on higher densities of less saturated species. At least, one can simply assume that the smaller particle is featured by the lower ionization rate coefficients because of the lower process cross-section and/or higher ionization threshold $\varepsilon_{iz}$. An increase in gas pressure at W = const results in decreasing ionization rate coefficients (because of the same change in $T_{e}$ and mean electron energy that provides decreasing fraction of electrons with ε > $\varepsilon_{iz}$) as well as accelerates the loss of positive ions in bulk plasma (because of increasing plasma electronegativity and negative ion density). As such, the decreasing tendency for $J_{+}=f\left(p\right)$ is probably due to corresponding changes in both $n_{+}$ and ion Bohm velocity.
- The negative dc bias at constant bias power (Figure 4c) always shows opposite trends compared with $J_{+}$. The reason is that the positive ion flux partly compensates for the negative charge produced by bias source. In both gas systems, the growth of ion flux vs. input power overlaps the weaker decrease in $-U_{dc}$ and provides the intensification of the physical etching pathway. The last conclusion directly follows from the change in $\sqrt{\varepsilon_{i}}\Gamma_{+}$ values shown in Figure 4d. Accordingly, the combination of decreasing ion flux and the nearly constant $-U_{dc}$ with increasing gas pressure lowers the ion bombardment intensity and thus, suppresses the physical etching pathway. Similar effects have been reported for various gas systems [2,5,6].

When summarizing the above data, one can conclude that both gas mixtures have no principal differences in respect to the efficiency of electron-impact processes. Really, though the C_6_F_12_O + Ar plasma exhibits higher $T_{e}$ values in the range of W > 400 W, it is formally compensated by lower electron densities, as follows from corresponding dissimilarities in $n_{+}$. This allows one to assume close dissociation frequencies ($k_{dis}n_{e}$, where $k_{dis}$ is the dissociation rate coefficient) for one and the same species as well as to attribute peculiarities of corresponding gas phase compositions to various dissociation pathways for original C_6_F_12_O and CF_4_ molecules. As for the intensity of ion bombardment that determines rates of ions-driven heterogeneous processes, some evident differences do exist. As can be seen from Figure 4d, the gap between $\sqrt{\varepsilon_{i}}\Gamma_{+}$ values becomes noticeable at W > 400 W and reaches about two times at W = 600 W. Obviously, the last effect is mainly due to the lower ion flux in the C_6_F_12_O + Ar plasma, as can be understood from Figure 4b. The similar difference in ion fluxes was also mentioned in Ref. [27] for oxygen containing CF_4_ and C_6_F_12_O plasmas under the close range of processing conditions. Therefore, such stable feature may provide an advance in respect to the low-damage etching.

**Figure 5:**
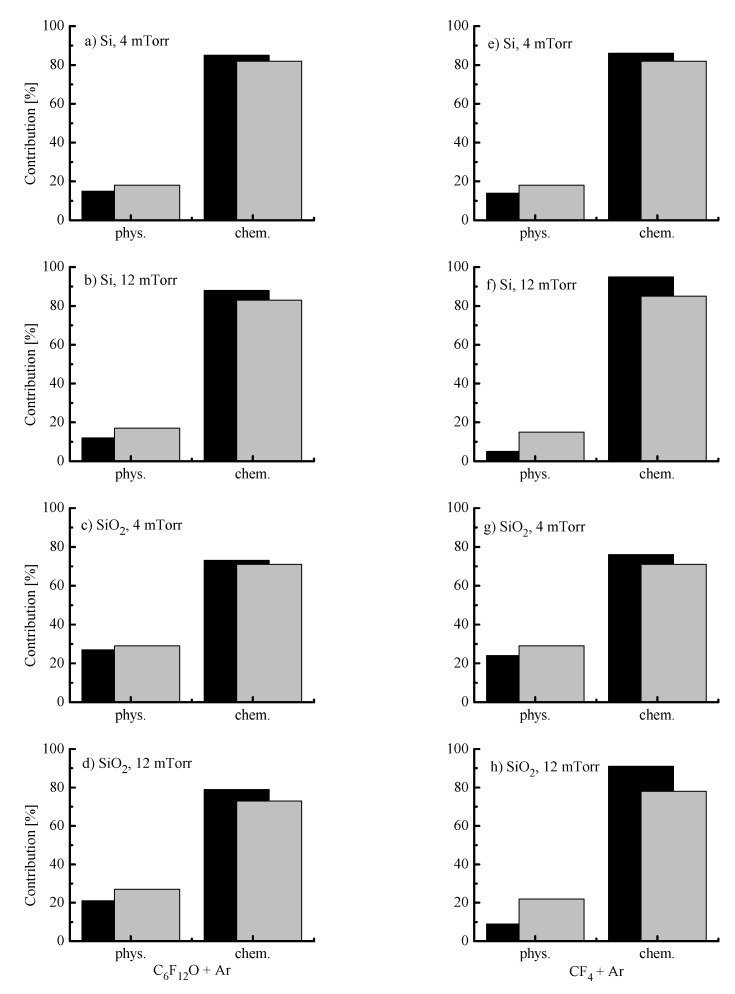
Relative contributions of physical (sputter etching) and chemical (ion-assisted chemical reaction) etching pathways to Si (**a**,**b**,**e**,**f**) and SiO_2_ (**c**,**d**,**g**,**h**) etching rates at W = 200 W (black bars) and 600 W (grey bars).

In order to compare contributions of $R_{phys}$ to measured etching rates, one can refer for direct experimental data on corresponding sputtering yields [38,39]. From these works, one can conclude that (a) in the range of $\varepsilon_{i}$ < 600 eV, sputtering yields for Si and SiO_2_ are close enough to be characterized by an average common value of $Y_{S}$; and (b) actual ion energy ranges of 360–460 eV (for C_6_F_12_O + Ar plasma) and 355–510 eV (for CF_4_ + Ar plasma) correspond to $Y_{S}$ ~0.35–0.44 atom/ion and 0.35–0.48 atom/ion, respectively. Accordingly, this allows one to calculate $R_{phys}\approx Y_{S}\Gamma_{+}$ (see Section 2.2) as well as to find $R_{chem}=R-R_{phys}$. Calculations showed that the rate of sputter etching always increases with increasing input power (for example, 11–31 mn/min for C_6_F_12_O + Ar plasma and 12–51 mn/min for CF_4_ + Ar plasma at p = 8 mTorr) and decreases toward higher gas pressures (for example, 25–21 nm/min for C_6_F_12_O + Ar plasma and 34–29 mn/min for CF_4_ + Ar plasma at W = 400 W). Though the C_6_F_12_O + Ar plasma provides lower $R_{phys}$ values (as was predicted by Figure 4d), it exhibits generally higher $R_{phys}/R$ ratios (Figure 5) due to lower total etching rates compared with those for CF_4_ + Ar. Another important finding is that both gas systems surely satisfy the rule of $R_{chem}$ *>>* $R_{phys}$ which means that the change in any measured etching rate is mainly controlled by $R_{chem}$ (Figure 2c,d). The contribution of $R_{phys}$ appears to be higher for SiO_2_ at lower pressures and higher input powers but does not exceed 30% in its maximum value. From the comparison of Figure 2c,d and Figure 4d, it can be seen also that the change of $R_{chem}$ vs. gas pressure contradicts with that for ion energy flux. This surely confirms that the dominant etching mechanism for Si and SiO_2_ in both gas systems is the ion-assisted chemical reaction which appears in either pure reaction-rate-limited ($\gamma_{R}$ ≈ const) or transitional ($\gamma_{R}$ ≠ const) regime. The last case assumes that the whole process rate is generally controlled by F atom flux while the effective probability for Si(s.) + xF → SiF_x_ reaction may be sensitive to the ion momentum flux through ion-stimulated desorption of reaction products, oxide bond breaking, and/or fluorocarbon film thickness. That is why, the understanding of the real situation requires the analysis of correlations between $R_{chem}$ and the fluorine atom flux. As such, the data on F atom densities are strongly required.

**Figure 6:**
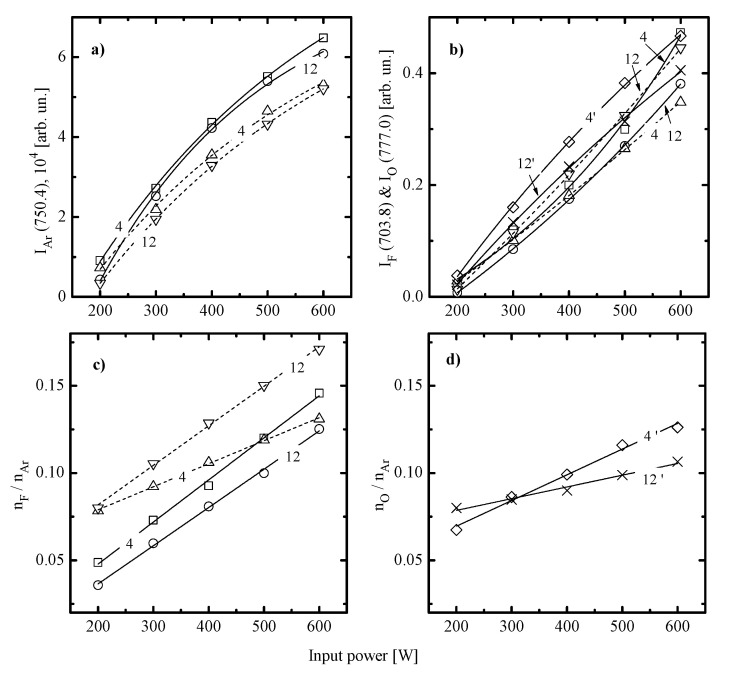
Optical emission spectroscopy (OES) diagnostics of 50% C_6_F_12_O + 50% Ar (solid lines) and 50% CF_4_ + 50% Ar (dashed lines) plasmas: (**a**) measured emission intensity for Ar 750.4 nm line; (**b**) measured emission intensities for F 703.8 nm and O 777.0 nm lines; (**c**) evaluated $n_{F}/n_{Ar}$ density ratio; and (**d**) evaluated $n_{O}/n_{Ar}$ density ratio. Numerical labels on curves mean the gas pressure in mTorr while the «’» mark at the corresponding label points out on oxygen-related data).

Figure 6 represents results of plasma diagnostics by optical emission spectroscopy (OES). From Figure 6a,b, it can be seen that an increase in input power at p = const always causes the faster growth in emission intensities for F (703.8 nm) and O (777.0 nm) lines compared with that for the Ar (750.4 nm) line. Such situation provides an increase in both F/Ar and O/Ar intensity ratios and thus, under the condition of $C_{act}^{X}$ ≈ const [35], directly corresponds to similar change in $n_{F}/n_{Ar}$ and $n_{O}/n_{Ar}$ (Figure 5c). Taking into account the nearly constant $n_{Ar}$ vs. input power, one can surely speak about linear increase in F atom densities toward higher input powers in both gas systems. Such phenomenon is in good agreement with numerous published data obtained by both plasma diagnostics and modeling for CF_4_ + Ar plasma (see, for example, Refs. [40,41]). The similar conclusion follows from Figure 6d in respect to O atom density in the C_6_F_12_O + Ar plasma. As for the effect of gas pressure, one must remember that the range of 4–12 mTorr provides the nearly three-fold increase in Ar density in a feed gas. That is, even a bit lower-running $n_{F}/n_{Ar}$ curve at 12 mTorr for C_6_F_12_O + Ar plasma in Figure 6c also corresponds to an increase in $n_{F}$ toward higher pressures. Therefore, influence of gas pressure on F atom density is also quite similar for both gas systems. It is important to note that similar slopes for $n_{F}/n_{Ar}$ curves at p = 12 mTorr in C_6_F_12_O + Ar and CF_4_ + Ar plasmas generally correspond to similar changes in electron-impact kinetics, as was mentioned above. The faster growth in $n_{F}/n_{Ar}$ ratio in C_6_F_12_O + Ar plasma at p = 4 mTorr contradicts this rule but exhibits the correlation with faster changes in $n_{O}/n_{Ar}$ ratio and O atom density. Probably, this suggests that the formation kinetics for F atoms at lower pressures is contributed by reactions with a participation of oxygen, such as CF_x_ + O/O(^1^D) → CF_x-1_O + F. 

When making a comparison between data of Figure 2 and Figure 6, the basic features of ion-assisted chemical reaction in C_6_F_12_O + Ar and CF_4_ + Ar gas systems may be suggested as follows:

(1) In both gas systems, the behavior of $R_{chem}$ qualitatively correlates with the corresponding trend of F atom density while lower Si and SiO_2_ etching rates in the C_6_F_12_O + Ar plasma generally correspond to lower $n_{F}$ values. This directly points to a similar limiting stage in a form of Si(s.) + xF → SiF_x_ reaction as well as probably means the non-principal influence of fluorocarbon polymer film on the etching kinetics.
(2) In both gas systems, the change in $R_{chem}$ appears to be quantitatively different than that for F atom density. In particular, the CF_4_ + Ar plasma at the low-pressure represents the special case when $R_{chem}$ for both Si and SiO_2_ increases faster compared with F atom density. Such situation corresponds to an increase in effective reaction probability that correlates with the behavior of $\sqrt{\varepsilon_{i}}\Gamma_{+}$. This allows one to suggest that, under the given set of processing conditions, an increase in ion momentum flux accelerates chemical reaction. At the same time, the high-pressure CF_4_ + Ar plasma, as well as the C_6_F_12_O + Ar plasma at any pressure within 4–12 mTorr, demonstrate the slower increase in $R_{chem}$ compared with F atom density. Accordingly, one can speak about decreasing effective reaction probability that contradicts with changes in ion momentum fluxes. In order to explain this phenomenon, one can simply suggest an increase in the amount of deposited polymer (which can be a true if the flux of polymerizing radicals growths faster compared with $\sqrt{\varepsilon_{i}}\Gamma_{+}$) or heterogeneous reactions with a participation of oxygen atoms. The last mechanism may suppress $\gamma_{R}$ in C_6_F_12_O + Ar plasma through the formation of oxide bonds and blocking of adsorption sites for F atoms.
(3) The situation $\gamma_{R}$ ≠ const obtained for all processing gases and conditions at constant surface temperature generally means the sensitivity of $\gamma_{R}$ to gas-phase plasma parameters, such as particle and/or energy fluxes. Here, though the main trend for $R_{chem}$ is determined by the F atom flux, the change in $\gamma_{R}$ affects the slope and/or the shape of corresponding curve and thus, provides the ability for additional process control. As such, the understanding of factors influencing $\gamma_{R}$ is the key point for understanding the etching mechanism itself.

Additional information on heterogeneous reaction pathways may principally be derived from XPS data for F(1s) shown in Figure 3e,f. First, the difference in intensities for F(1s)-C peaks in Figure 3e,f is in agreement with those for C(1s)-F from Figure 3a,b. This fact confirms a bit higher polymerizing ability of C_6_F_12_O + Ar plasma as well as supports the earlier-made conclusion on a non-principal dissimilarity of C_6_F_12_O + Ar and CF_4_ + Ar gas system in respect to this parameter. It can be concluded also that the polymer film deposited in C_6_F_12_O + Ar plasma is featured by the different chemical structure. At least, the peak at ~690.6 eV in Figure 3e may be attributed to F atoms in the polymer chain with multi-carbon species [41,42]. Secondly, the difference in intensities for F(1s)-Si peaks indicate the higher amount of residual SiF_x_ compounds after the treatment in C_6_F_12_O + Ar plasma. In the light of lower etching rates, such situation may take place only if the given gas system provides the worse removal of etching products. Obviously, the last feature cannot be directly associated with lower $\sqrt{\varepsilon_{i}}\Gamma_{+}$ values (Figure 4d), since the chemical reaction between Si and F atoms leads to the formation of high-volatile SiF_4_ [4,5]. In our opinion, the oxygen containing plasma may result in the formation of lower volatile SiOF_x_ compounds that can be gasified only by the ion-stimulated desorption. At least, the similar phenomenon has been mentioned for the etching of silicon in Cl_2_- and HBr-based plasmas in the presence of oxygen [43,44]. Quite close intensities for F(1s)-O peaks do not contradict with the above suggestion if to assume that oxygen is connected by a double bond only with the Si atom. 

**Figure 7:**
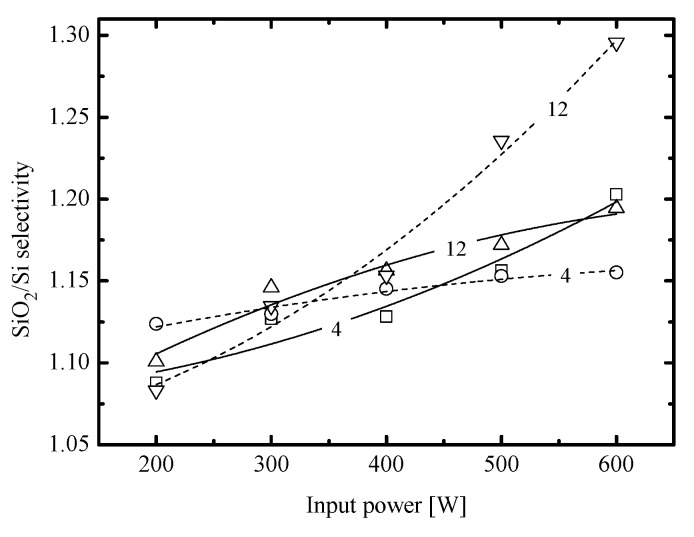
SiO_2_/Si etching selectivity in 50% C_6_F_12_O + 50% Ar (solid lines) and 50% CF_4_ + 50% Ar (dashed lines) plasmas. Numerical labels on curves mean the gas pressure in mTorr.

One more issue which seems to be worth the brief discussion is the SiO_2_/Si etching selectivity. From Figure 7, it can be seen that the SiO_2_/Si etching selectivity in C_6_F_12_O + Ar plasma shows the weak growth with increasing input power as well as exhibits the low sensitivity to gas pressure. Both gas systems demonstrate the quite similar situation at the low pressure end ($R_{SiO_{2}}/R_{Si}$ ~1.16 for the CF_4_-based plasma and ~1.21 for the C_6_F_12_O-based plasma at 4 mTorr and 500 W), but exhibits the noticeable difference for higher pressures and input powers. In particular, the maximum $R_{SiO_{2}}/R_{Si}$ value in the CF_4_-based plasma at 12 mTorr reaches 1.3 while the C_6_F_12_O-based plasma produces only ~1.2. The simplest explanation of this fact may be connected with differences in polymer film thicknesses. Probably, the combination of high pressure and input power creates favorable conditions for increasing flux of polymerizing radicals and polymer deposition rates. At the same time, the C_6_F_12_O-based plasma is also characterized by increasing O atom flux that accelerates the chemical etching rate of a polymer film. As such, the latter appears to be thinner and has the much lower impact on Si and SiO_2_ etching kinetics. In our opinion, this suggestion does not contradict with Figure 2 which indicates the rather close amounts of deposited polymer for both gas systems. The reason is that conditions of Figure 3 relate to an intermediate case (8 mTorr and 400 W) where differences in $h_{pol}$ are not sufficient yet. Accordingly, corresponding points in Figure 7 are also very close to each other. Therefore, the effect of own oxygen limits the ability of gas pressure and input power to adjust the SiO_2_/Si etching selectivity in C_6_F_12_O-based plasmas.

Finally, we would like to note that the above analysis is only a primary step that just provides reasonable explanations for etching phenomenology. Obviously, more accurate conclusions in respect to etching mechanisms assume, at least, the more detailed study of the C_6_F_12_O + Ar gas system with obtaining quantitative data on densities of F atoms, O atoms, and polymerizing radicals. Since corresponding values for CF_4_ + Ar plasma are well-known from published works (or may be easily obtained by plasma modeling with the well-adjusted kinetic scheme [17,32,33,34,40]), one will be able to compare real particle fluxes as well as to discuss contributions of various physical or chemical processes to the change of effective reaction probabilities. At the same time, the simplified approach used in this study evidently illustrates the principal features which may be important when making a choice between two gas systems for the purpose of given etching process. Particularly, the principal finding in this study is that the C_6_F_12_O gas with low global warming potential may be the almost equivalent (with some limitations in respect to etching selectivity) replacement for CF_4_ in the reactive-ion etching of Si and SiO_2_. At the same time, lower absolute etching rates in the C_6_F_12_O-based plasma provide the condition for better process control. In the light of the very poor knowledge on the C_6_F_12_O + Ar plasma chemistry, such information may be a real value for future progress in dry etching technology.

## 4. Conclusions

This work reports on the comparative study of C_6_F_12_O + Ar and CF_4_ + Ar gas chemistries in respect to Si and SiO_2_ reactive-ion etching processes under the condition of low input power mode. Si and SiO_2_ etching rates in both gas systems were measured as functions of input power (200–600 W that corresponds to ~0.02–0.06 W/cm^3^) and gas pressure (4–12 mTorr). Plasma diagnostics by double Langmuir probe and optical emission spectroscopy indicated principal similarities of C_6_F_12_O + Ar and CF_4_ + Ar plasmas in respect to the influence of processing conditions on physical plasma parameters (electron temperature, ion current density, plasma density, and ion momentum flux), electron-impact kinetics, and F atom density. In addition, the X-ray photoelectron spectroscopy showed no principal differences in corresponding polymerization abilities. The analysis of these data allowed one to compare performances of given gas systems in respect to the reactive-ion etching of Si and SiO_2_ as well as to associate features of corresponding etching kinetics with those for gas-phase plasma characteristics. It was shown that etching processes of both materials in both gas systems (a) have no ions-driven limiting stages; (b) are mainly provided by the ion-assisted chemical reaction controlled by the F atom flux; and (c) are characterized by the processing-condition-dependent (in other words—effective) reaction probability. Such situation assumes no principal differences in etching regimes. The final conclusion was that the C_6_F_12_O gas with low global warming potential may be the almost equivalent replacement for CF_4_ in the reactive-ion etching of Si and SiO_2_. The features of C_6_F_12_O-based plasma are a bit lower SiO_2_/Si etching selectivity at higher pressures as well as lower absolute etching rates. The last fact provides the opportunity for better process control.

## Data Availability

Data sharing not available.

## References

[1] Sze S.M. (1988). VLSI Technology.

[2] Sugano T. (1990). Applications of Plasma Processes to VLSI Technology.

[3] Rooth J.R. (1995). Industrial Plasma Engineering.

[4] Wolf S., Tauber R.N. (2000). Silicon Processing for the VLSI Era.

[5] Lieberman M.A., Lichtenberg A.J. (2005). Principles of Plasma Discharges and Materials Processing.

[6] Coburn J.W. (1982). Plasma Etching and Reactive Ion Etching.

[7] Stoffels W.W., Stoffels E., Tachibana K. (1998). Polymerization of fluorocarbons in reactive ion etching plasmas. J. Vac. Sci. Technol. A.

[8] Schaepkens M.M., Standaert T.E.F.M., Rueger N.R., Sebel P.P., Oehrlein G.G., Cook J.M. (1999). Study of the SiO_2_-to-Si_3_N_4_ etch selectivity mechanism in inductively coupled fluorocarbon plasmas and a comparison with the SiO2-to-Si mechanism. J. Vac. Sci. Technol. A.

[9] Standaert T.E.F.M., Hedlund C., Joseph E.A., Oehrlein G.S., Dalton T.J. (2004). Role of fluorocarbon film formation in the etching of silicon, silicon dioxide, silicon nitride, and amorphous hydrogenated silicon carbide. J. Vac. Sci. Technol. A.

[10] Lee H.K., Chung K.S., Yu J.S. (2009). Selective Etching of Thick Si_3_N_4_, SiO_2_ and Si by Using CF_4_/O_2_ and C_2_F_6_ Gases with or without O_2_ or Ar Addition. J. Korean Phys. Soc..

[11] Kastenmeier B.E.E., Matsuo P.J., Oehrlein G.S. (1999). Highly selective etching of silicon nitride over silicon and silicon dioxide. J. Vac. Sci. Technol. A.

[12] Lele C., Liang Z., Linda X., Dongxia L., Hui C., Tod P. (2009). Role of CF_2_ in the etching of SiO_2_, Si_3_N_4_ and Si in fluorocarbon plasma. J. Semicond..

[13] Matsui M., Tatsumi T., Sekine M. (2001). Relationship of etch reaction and reactive species flux in C_4_F_8_/Ar/O_2_ plasma for SiO_2_ selective etching over Si and Si_3_N_4_. J. Vac. Sci. Technol..

[14] Li X., Ling L., Hua X., Fukasawa M., Oehrlein G.S., Barela M., Anderson H.M. (2003). Effects of Ar and O_2_ additives on SiO_2_ etching in C_4_F_8_-based plasmas. J. Vac. Sci. Technol..

[15] Li X., Hua X., Ling L., Oehrlein G.S., Wang Y., Anderson H.M. (2003). Characteristics of C_4_F_8_ plasmas with Ar, Ne, and He additives for SiO_2_ etching in an inductively coupled plasma (ICP) reactor. J. Vac. Sci. Technol..

[16] Sankaran A., Kushner M.J. (2005). Etching of porous and solid SiO_2_ in Ar/c-C_4_F_8_, O2/c-C_4_F_8_ and Ar/O_2_/c-C_4_F_8_ plasmas. J. Appl. Phys..

[17] Lee J., Efremov A., Yeom G.Y., Lim N., Kwon K.H. (2015). Application of Si and SiO_2_ Etching Mechanisms in CF_4_/C_4_F_8_/Ar Inductively Coupled Plasmas for Nanoscale Patterns. J. Nanosci. Nanotechnol..

[18] Lide D.R. (1998). Handbook of Chemistry and Physics.

[19] Van Roosmalen A.J., Baggerman J.A.G., Brader S.J.H. (1991). Dry Etching for VLSI.

[20] Tran-Quinn T., Lakritz M. Unsaturated Fluorocarbons in the Etching Process, Environmental Benefit, Technical Hurdles. Proceedings of the 2008 IEEE/SEMI Advanced Semiconductor Manufacturing Conference.

[21] Muhle J., Ganesan A.L., Miller B.R., Salameh P.K., Harth C.M., Greally B.R., Rigby M., Porter L.W., Steele L.P., Trudinger C.M. (2010). Perfluorocarbons in the global atmosphere: Tetrafluoromethane, hexafluoroethane, and octafluoro-propane. Atmos. Chem. Phys..

[22] Kiehlbauch M.W., Graves D.B. (2001). Temperature resolved modeling of plasma abatement of perfluorinated compounds. J. Appl. Phys..

[23] Bolaji B., Huan Z. (2013). Ozone depletion and global warming: Case for the use of natural refrigerant—A review. Renew. Sustain. Energy Rev..

[24] Krishnan N., Smati R., Raoux S., Dornfeld D. Alternatives to reduce perfluorinated compound (PFC) emissions from semi-conductor dielectric etch processes: Meeting environmental commitments while minimizing costs. Proceedings of the International Symposium on Electronics and the Environment (IEEE).

[25] Mocella M.T. (1996). PFC Emission Control Options for Plasma Processing Tools: A Current Assessment.

[26] Tian S., Zhang X., Wang Y., Rao X., Ye F., Li Y., Xiao S. (2019). Partial discharge characteristics of C_6_F_12_O/CO_2_ mixed gas at power frequency AC voltage. AIP Adv..

[27] Lee J., Nam Y., Lee J., Lee H.W., Kwon K.-H. (2020). Etching characteristics of thin SiON films using a liquefied perfluorocarbon precursor of C_6_F_12_O with a low global warming potential. Plasma Sci. Technol..

[28] Veselov D.S., Bakun A.D., Voronov Y.A. (2016). Reactive ion etching of silicon using low-power plasma etcher. J. Phys. Conf. Ser..

[29] Ashraf M., Sundararajan S.V., Grenc G. (2017). Low-power, low-pressure reactive-ion etching process for silicon etching with ver-tical and smooth walls for mechanobiology application. J. Micro Nanolith. MEMS MOEMS.

[30] Lee J., Kwon K.H., Efremov A. (2018). On the Relationships Between Plasma Chemistry, Etching Kinetics and Etching Residues in CF_4_ + C_4_F_8_ + Ar and CF_4_ + CH_2_F_2_ + Ar Plasmas with Various CF_4_/C_4_F_8_ and CF_4_/CH_2_F_2_ Mixing Ratios. Vacuum.

[31] Shun’ko E.V. (2008). Langmuir Probe in Theory and Practice.

[32] Efremov A., Lee J., Kim J. (2017). On the Control of Plasma Parameters and Active Species Kinetics in CF_4_+ O_2_+ Ar Gas Mixture by CF_4_/O_2_ and O_2_/Ar Mixing Ratios. Plasma Chem. Plasma Process..

[33] Chun I., Efremov A., Yeom G.Y., Kwon K.H. (2015). A comparative study of CF_4_/O_2_/Ar and C_4_F_8_/O_2_/Ar plasmas for dry etching applications. Thin Solid Films.

[34] Lim N., Efremov A., Kwon K.H. (2019). Gas-phase chemistry and etching mechanism of SiN_x_ thin films in C_4_F_8_ + Ar inductively coupled plasma. Thin Solid Films.

[35] Lopaev D.V., Volynets A.V., Zyryanov S.M., Zotovich A.I., Rakhimov A.T. (2017). Actinometry of O, N and F atoms. J. Phys. D Appl. Phys..

[36] Winters H.F. (1983). Surface processes in plasma-assisted etching environments. J. Vac. Sci. Technol. B Microelectron. Nanometer Struct..

[37] Gray D.C., Tepermeister I., Sawin H.H. (1993). Phenomenological modeling of ion-enhanced surface kinetics in fluorine-based plasma etching. J. Vac. Sci. Technol..

[38] Zalm P.C. (1983). Energy dependence of the sputtering yield of silicon bombarded with neon, argon, krypton, and xenon ions. J. Appl. Phys..

[39] Seah M.P., Nunney T.S. (2010). Sputtering yields of compounds using argon ions. J. Phys. D Appl. Phys..

[40] Kimura T., Ohe K. (2002). Model and probe measurements of inductively coupled CF_4_ discharges. J. Appl. Phys..

[41] Sasaki K., Kawai Y., Kadota K. (1999). Determination of fluorine atom density in reactive plasmas by vacuum ultraviolet absorption spectroscopy at 95.85 nm. Rev. Sci. Instrum..

[42] (2012). NIST X-Ray Photoelectron Spectroscopy Database.

[43] Cunge G., Kogelschatz M., Joubert O., Sadeghi N. (2005). Plasma–wall interactions during silicon etching processes in high-density HBr/Cl_2_/O_2_ plasmas. Plasma Sources Sci. Technol..

[44] Tinck S., Boullart W., Bogaerts A. (2011). Modeling Cl_2_/O_2_/Ar inductively coupled plasmas used for silicon etching: Effects of SiO_2_ chamber wall coating. Plasma Sources Sci. Technol..

